# Chloroquine inhibits Ca^2+^ permeable ion channels-mediated Ca^2+^ signaling in primary B lymphocytes

**DOI:** 10.1186/s13578-017-0155-5

**Published:** 2017-05-23

**Authors:** Yi-Fan Wu, Ping Zhao, Xi Luo, Jin-Chao Xu, Lu Xue, Qi Zhou, Mingrui Xiong, Jinhua Shen, Yong-Bo Peng, Meng-Fei Yu, Weiwei Chen, Liqun Ma, Qing-Hua Liu

**Affiliations:** Institute for Medical Biology & Hubei Provincial Key Laboratory for Protection and Application of Special Plants in Wuling Area of China, College of Life Sciences, South Central University for Nationalities, Wuhan, 430074 China

**Keywords:** B cells, Ca^2+^, Chloroquine, IP_3_R, TRPC3 channels, STIM/Orai channels

## Abstract

**Background:**

Chloroquine, a bitter tastant, inhibits Ca^2+^ signaling, resulting in suppression of B cell activation; however, the inhibitory mechanism remains unclear.

**Results:**

In this study, thapsigargin (TG), but not caffeine, induced sustained intracellular Ca^2+^ increases in mouse splenic primary B lymphocytes, which were markedly inhibited by chloroquine. Under Ca^2+^-free conditions, TG elicited transient Ca^2+^ increases, which additionally elevated upon the restoration of 2 mM Ca^2+^. The former were from release of intracellular Ca^2+^ store and the latter from Ca^2+^ influx. TG-induced release was inhibited by 2-APB (an inhibitor of inositol-3-phosphate receptors, IP_3_Rs) and chloroquine, and TG-caused influx was inhibited by pyrazole (Pyr3, an inhibitor of transient receptor potential C3 (TRPC3) and stromal interaction molecule (STIM)/Orai channels) and chloroquine. Moreover, chloroquine also blocked Ca^2+^ increases induced by the engagement of B cell receptor (BCR) with anti-IgM.

**Conclusions:**

These results indicate that chloroquine inhibits Ca^2+^ elevations in splenic B cells through inhibiting Ca^2+^ permeable IP_3_R and TRPC3 and/or STIM/Orai channels. These findings suggest that chloroquine would be a potent immunosuppressant.

## Background

Chloroquine is a bitter tastant [1–4], which was used to treat malaria [5] and immune-related diseases such as rheumatic disease, systemic lupus erythematosus [6], early-stage AIDS [7] and chronic graft-versus-host disease [8]. Moreover, it inhibits Ia molecule biosynthesis [9] and CpG DNA-induced protection [10] in B cells. These results imply that chloroquine might be an immunosuppressant of B cell activation. Cytosolic Ca^2+^ increases play an important role in B cell development [9], survival [11], activation [12], and differentiation [13, 14], cytokine production [12] and cell death [15]. Ca^2+^ increases in B cells are induced by antigen or anti-B cell receptor (BCR) ligation. BCR antibodies bind to the BCR, resulting in the phosphorylation of tyrosine in phospholnositide-specific phospholipase C (PLC). The phosphorylated PLC catalyzes phosphatidylinositol-4, 5-bisphosphate (PIP_2_) into diacylglycerol (DAG) and inositol-1,4,5-trisphosphate (IP_3_). IP_3_ binds to IP_3_Rs located on the surface of the endoplasmic reticulum (ER). The activated IP_3_Rs then mediate Ca^2+^ release from the ER, leading to increases in intracellular Ca^2+^ [15, 16]. In addition, Ca^2+^ increases can also be induced by thapsigargin (TG) [17]. However, whether and how bitter tastant chloroquine inhibits Ca^2+^ increases in B cells remains unclear.

In this study, we found that chloroquine inhibited Ca^2+^ increases induced by TG and BCR engagement with anti-IgM through inhibiting Ca^2+^ permeable ion channels.

## Results

### Chloroquine decreases TG-induced increases of intracellular Ca^2+^

**Fig. 1:**
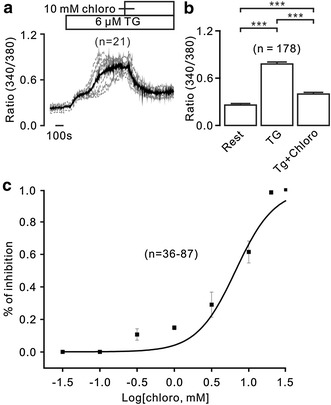
Chloroquine (chloro) blocks increases of Ca^2+^. **a** TG induced steady increases of Ca^2+^ in splenic primary B lymphocytes, which were blocked by chloro. The *bold line* represents the average values. **b** The average Ca^2+^ levels from 178 cells. **c** Dose-dependent inhibition of chloro on TG-induced Ca^2+^ increases. ***p < 0.001. These results indicate that chloro attenuates TG-induced elevations of Ca^2+^

In this study, we sought to investigate the effect of chloroquine on intracellular Ca^2+^ in primary B lymphocytes from mouse spleens. As shown in Fig. 1a and b, the increases of Ca^2+^ were induced by TG, an inhibitor of the ER Ca^2+^ ATPase, which were inhibited by chloroquine. The dose-relationship of inhibition is shown in Fig. 1c. The IC_50_ was 9.3 ± 0.7 mM (Fig. 1c). These results indicate that chloroquine inhibits TG-induced elevations of cytosolic Ca^2+^.

### Mechanism of chloroquine-caused inhibition on TG-induced Ca^2+^ increases

**Fig. 2:**
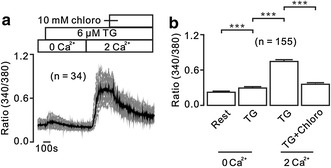
Chloro inhibits extracellular Ca^2+^ influx. **a** TG induced small transient increases of Ca^2+^ under Ca^2+^-free conditions (0 mM Ca^2+^ and 0.5 mM EGTA). Following the restoration of 2 mM Ca^2+^, large sustained elevations occurred and were declined by chloro. **b** The average Ca^2+^ levels from 155 cells. ***p < 0.001. These results indicate that chloro inhibits extracellular Ca^2+^ influx

To study whether chloroquine inhibits extracellular Ca^2+^ entry, we performed the following experiments. Intracellular Ca^2+^ store was first depleted by TG under Ca^2+^-free conditions (0 mM Ca^2+^ and 0.5 mM EGTA), which resulted in transient increases of Ca^2+^. Ca^2+^ (2 mM) was then restored in the extracellular solutions, which induced additional increases and were markedly blocked by chloroquine (Fig. 2a). The Ca^2+^ levels in 155 cells were summarized (Fig. 2b). These data indicate that chloroquine inhibits Ca^2+^ influx.

**Fig. 3:**
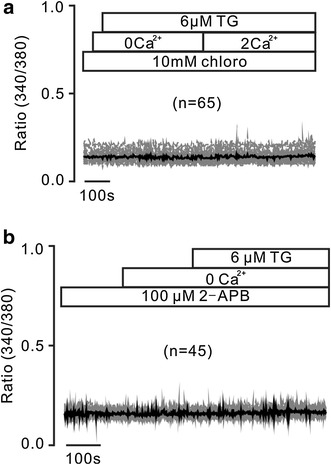
Chloro blocks TG-induced Ca^2+^ release by inhibiting IP_3_Rs on the ER membrane. **a** After cells were incubated with chloro, neither TG nor the addition of 2 mM Ca^2+^ induced increases of Ca^2+^. These experiments were performed in 241 cells. These data suggest that chloro inhibits TG-induced Ca^2+^ release. **b** Following cells were incubated with 2-APB, an IP_3_R blocker, TG failed to induce increases of Ca^2+^ under Ca^2+^-free conditions (0 mM Ca^2+^ and 0.5 mM EGTA). These experiments were conducted in 129 cells. These data suggest that 2-APB inhibits TG-induced Ca^2+^ release from intracellular Ca^2+^ stores by inhibiting IP_3_Rs on the ER membrane

We next investigated whether chloroquine inhibits TG-induced intracellular Ca^2+^ release. Under Ca^2+^-free conditions (0 mM Ca^2+^ and 0.5 mM EGTA), the incubation of chloroquine abolished TG-induced transient Ca^2+^ increases and the restoration of 2 mM Ca^2+^-caused additional elevations (Fig. 3a). This phenomenon was observed in 241 cells. These results indicate that chloroquine blocks Ca^2+^ release from the ER.

We then studied which Ca^2+^ release pathway was inhibited by chloroquine. It has known that Ca^2+^ release is mainly mediated by IP_3_Rs and RyRs. 2-APB is a blocker of IP_3_R [20]. As shown in Fig. 3b, 2-APB abrogated TG-induced increases of Ca^2+^ under Ca^2+^-free conditions (0 mM Ca^2+^ and 0.5 mM EGTA). These inhibitions were observed in 129 cells. These results indicate that chloroquine inhibits IP_3_R-mediated Ca^2+^ release from the ER.

**Fig. 4:**
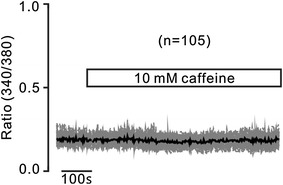
Caffeine fails to trigger increases of Ca^2+^. Caffeine did not trigger increases of Ca^2+^ under 2 mM Ca^2+^ conditions. These were observed in 227 cells. These results suggest that these cells do not have functional RyRs

Next, we investigate the role of RyRs, since which mediate Ca^2+^ release from the ER [21]. Cells were stimulated with caffeine, a selective activator of RyRs, which failed to increase Ca^2+^ (Fig. 4). These results indicate that these cells have no functional RyRs, suggesting that RyRs do not contribute to TG-induced Ca^2+^ release.

**Fig. 5:**
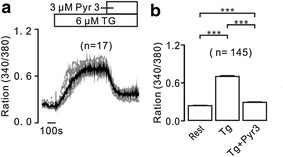
Pyr3 attenuates TG-induced Ca^2+^ elevations. **a** TG-induced sustained increases of Ca^2+^ were reduced by Pyr3, a selective inhibitor of TRPC3 and STIM/Orai channels. **b** The average values from 145 cells. ***p < 0.001. These results suggest that TRPC3 and/or STIM/Orai channel mediate TG-induced Ca^2+^ increases

Chloroquine inhibits Ca^2+^ influx as shown in Fig. 2, we then studied the underlying mechanism. In previous studies, it has been found that chloroquine blocked TRPC3 and/or STIM/Orai channels, resulting in decreases of Ca^2+^ in airway smooth muscle cells [3, 18] and in murine CD4^+^ thymocytes [4]. As shown in Fig. 5, TG-induced increases were declined by Pyr3, an inhibitor of TRPC3 and/or STIM/Orai channels, suggesting that chloroquine inhibits TRPC3 and/or STIM/Orai channels-mediated Ca^2+^ influx.

### Chloroquine inhibits BCR engagement-induced Ca^2+^ elevations

**Fig. 6:**
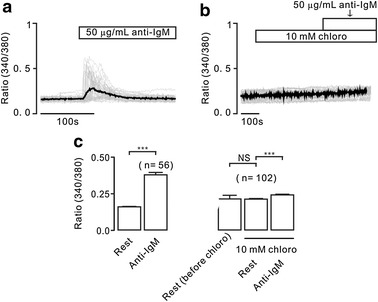
Chloro inhibits anti-IgM-induced Ca^2+^ increases. **a** Anti-IgM induced Ca^2+^ increases. **b** Chloro failed to affect the level of Ca^2+^, however, inhibited anti-IgM-induced increases were inhibited by chloro. **c** The summary results. NS: *p* > 0.05; ***p < 0.001. These results indicate that chloro blocks BCR engagement-induced Ca^2+^ increases

We finally observed the effect of chloroquine on BCR engagement-induced Ca^2+^ increases. Our previous results indicate that the engagement of BCR with anti-IgM induces Ca^2+^ elevations in B cells [16]. As shown in Fig. 6, anti-IgM induced Ca^2+^ increases. However, such increases were potently inhibited by the incubation of chloroquine, although that the statistical results show that anti-IgM still induced a significant elevation. These results indicate that chloroquine inhibits BCR engagement-induced Ca^2+^ elevations.

## Discussion

In the present study, our results indicate that chloroquine depresses Ca^2+^ increases induced by TG and anti-IgM via inhibiting the intracellular Ca^2+^ release mediated by IP_3_R channels and inhibiting the extracellular Ca^2+^ influx mediated by TRPC3 and/or STIM/Orai channels.

The aim of this study was to investigate whether chloroquine inhibits increases of Ca^2+^ and the underlying mechanism. We found that chloroquine inhibited increases of Ca^2+^ induced by TG (Fig. 1). To define the inhibitory mechanism, we investigated the pathways that mediated TG-induced Ca^2+^ elevations. Intracellular Ca^2+^ increases will be mediated by Ca^2+^ permeation channels on the plasma and the ER membrane [22]. These have been demonstrated by the results that under Ca^2+^-free conditions, TG induced transient increases and followed by additional elevations upon the restoration of 2 mM Ca^2+^ (Fig. 2), since the former resulted from release and the latter from influx. Moreover, the release was mediated by IP_3_R in the ER membrane (Fig. 3a, b), because that 2-APB blocked release-induced Ca^2+^ elevations. IP_3_Rs have three isoforms (IP_3_R1, IP_3_R2, and IP_3_R3), which can be inhibited by 2-APB [20]. While, the influx was mediated by TRPC3 and/or STIM/Orai channels in the plasma membrane. Because that Pyr3 (Fig. 5) blocked TG-induced sustained increases, which will be mediated by influx based on the results shown in Fig. 2. These data indicate that TG-induced intracellular Ca^2+^ elevations were mediated by above described ion channels, and which will be inhibited by chloroquine and then resulting in decreases. These results are consistent with previous findings that chloroquine blocks TRPC3 and/or STIM/Orai channels resulting in decreases in Ca^2+^ levels in smooth muscle cells [1, 3] and in murine CD4^+^ thymocytes [4]. In addition, BCR engagement frequently occurs in vivo, which then results in immunological responses. Therefore, we observed whether the engagement can induce Ca^2+^ increases and are blocked by chloroquine. The results show that chloroquine inhibited BCR engagement-induced Ca^2+^ increases (Fig. 6).

Ca^2+^ is a crucial second messenger that modulates many cellular processes in splenic B cells, such as cell differentiation and activation [14, 23]. Therefore, our data would indicate that chloroquine might be a potent inhibitor for B cells-mediated immunological responses and inflammations.

## Conclusions

Chloroquine inhibits Ca^2+^-permeable ion channels in the plasma and the ER membranes, resulting in decreases of Ca^2+^. These findings suggest that chloroquine would be an immunosuppressant.

## Methods

### Animals

6- to 8-week-old BALB/c male mice were purchased from the Hubei Provincial Center for Disease Control and Prevention, Wuhan, China. The mice were housed under controlled temperature (21–23 °C) and light (lights on between 08:00 and 20:00) conditions and were provided adequate water and food. All housing and experiments were performed in according with the Guide for the Institutional Animal Care and Use Committee of the South-Central University for Nationalities.

### Reagents

Fura-2 AM was purchased from Invitrogen (Eugene, OR, USA). Pyrazole-3 (Pyr3) and 2-Aminoethoxydiphenyl borate (2-APB) were purchased from Sigma-Aldrich (St. Louis, MO, USA). Thapsigargin (TG) was purchased from Cayman (Tallinn, Estonia). RPMI 1640 medium and fetal bovine serum (FBS) were purchased from Gibco (Rockville, MD, USA). Anti-B220-PE and anti-IgM antibodies were purchased from BD Pharmingen (San Diego, CA, USA). All of the other chemicals were purchased from Sinopharm Chemical Reagent Co. (Shanghai, China).

### Isolation of B cells

B lymphocytes were isolated from mouse spleens as previously described [16]. Briefly, after the animals were killed, the spleens were removed and placed in RPMI 1640 medium containing 10% FBS and 1 mM l-glutamine. The spleens were then gently teased apart with two G27 syringe needles. The non-cellular tissues were removed by filtering this preparation through a 70-μM nylon mesh. The cells were maintained at room temperature.

### Measurement of intracellular Ca^2+^

Intracellular Ca^2+^ was measured using fura-2 AM as previously described [18, 19]. The cells were loaded with 2.5 μM fura-2 AM. Paired 340/380 fluorescence images were acquired using a TILL imaging system (FEI Munich GmbH, Munich, Germany), and the fluorescence ratios represent the intracellular Ca^2+^ levels. The B cells were identified by anti-B220-PE.

### Data analysis and statistics

All of the data are presented as the mean ± SEM. The n values represent the number of cells. Unpaired Student’s *t* tests were performed to identify significant differences between the means. Differences with *p* < 0.05 were considered statistically significant.

## Abbreviations

TG: thapsigargin
IP_3_Rs: inositol-3-phosphate receptors
TRPC3: transient receptor potential C3
STIM: stromal interaction molecule
BCR: B cell receptor
PLC: phospholnositide-specific phospholipase C
PIP_2_: phosphatidylinositol-4, 5-bisphosphate
DAG: diacylglycerol
IP_3_: inositol-1,4,5-trisphosphate
ER: endoplasmic reticulum
2-APB: 2-aminoethoxydiphenyl borate
Pyr3: pyrazole-3
FBS: fetal bovine serum
RyRs: ryanodine receptors

## References

[1] Sai WB, Yu MF, Wei MY, Lu Z, Zheng YM, Wang YX, Qin G, Guo D, Ji G, Shen J, Liu QH (2014). Bitter tastants induce relaxation of rat thoracic aorta precontracted with high K(+). Clin Exp Pharmacol Physiol.

[2] Deshpande DA, Wang WC, McIlmoyle EL, Robinett KS, Schillinger RM, An SS, Sham JS, Liggett SB (2010). Bitter taste receptors on airway smooth muscle bronchodilate by localized calcium signaling and reverse obstruction. Nat Med.

[3] Zhang T, Luo XJ, Sai WB, Yu MF, Li WE, Ma YF, Chen W, Zhai K, Qin G, Guo D (2014). Non-selective cation channels mediate chloroquine-induced relaxation in precontracted mouse airway smooth muscle. PLoS ONE.

[4] Xu JC, Peng YB, Wei MY, Wu YF, Guo D, Qin G, Ji G, Shen J, Liu QH (2015). Chloroquine inhibits Ca(2 +) signaling in murine CD4(+) thymocytes. Cell Physiol Biochem.

[5] Gostner JM, Schrocksnadel S, Becker K, Jenny M, Schennach H, Uberall F, Fuchs D (2012). Antimalarial drug chloroquine counteracts activation of indoleamine (2,3)-dioxygenase activity in human PBMC. FEBS Open Bio..

[6] Wozniacka A, Lesiak A, Narbutt J, McCauliffe DP, Sysa-Jedrzejowska A (2006). Chloroquine treatment influences proinflammatory cytokine levels in systemic lupus erythematosus patients. Lupus..

[7] Paton NI, Goodall RL, Dunn DT, Franzen S, Collaco-Moraes Y, Gazzard BG, Williams IG, Fisher MJ, Winston A, Fox J (2012). Effects of hydroxychloroquine on immune activation and disease progression among HIV-infected patients not receiving antiretroviral therapy: a randomized controlled trial. JAMA.

[8] Gilman AL, Chan KW, Mogul A, Morris C, Goldman FD, Boyer M, Cirenza E, Mazumder A, Gehan E, Cahill R (2000). Hydroxychloroquine for the treatment of chronic graft-versus-host disease. Biol Blood Marrow Transplant.

[9] Nowell J, Quaranta V (1985). Chloroquine affects biosynthesis of Ia molecules by inhibiting dissociation of invariant (gamma) chains from alpha-beta dimers in B cells. J Exp Med.

[10] Yi AK, Peckham DW, Ashman RF, Krieg AM (1999). CpG DNA rescues B cells from apoptosis by activating NFkappaB and preventing mitochondrial membrane potential disruption via a chloroquine-sensitive pathway. Int Immunol.

[11] Dugas B, Calenda A, Delfraissy JF, Vazquez A, Bach JF, Galanaud P (1987). The cytosolic free calcium in anti-mu-stimulated human B cells is derived partly from extracellular medium and partly from intracellular stores. Eur J Immunol.

[12] Berridge MJ, Lipp P, Bootman MD (2000). The versatility and universality of calcium signalling. Nat Rev Mol Cell Biol.

[13] Dolmetsch RE, Lewis RS, Goodnow CC, Healy JI (1997). Differential activation of transcription factors induced by Ca^2+^ response amplitude and duration. Nature.

[14] Healy JI, Dolmetsch RE, Lewis RS, Goodnow CC (1998). Quantitative and qualitative control of antigen receptor signalling in tolerant B lymphocytes. Novartis Found Symp.

[15] Braun J, Sha’afi RI, Unanue ER (1979). Crosslinking by ligands to surface immunoglobulin triggers mobilization of intracellular 45Ca^2+^ in B lymphocytes. J Cell Biol.

[16] Liu QH, Liu X, Wen Z, Hondowicz B, King L, Monroe J, Freedman BD (2005). Distinct calcium channels regulate responses of primary B lymphocytes to B cell receptor engagement and mechanical stimuli. J Immunol..

[17] Okamoto Y, Furuno T, Hamano T, Nakanishi M (1995). Confocal fluorescence microscopy for studying thapsigargin-induced bivalent-cation entry into B cells. Biochem J..

[18] Badou A, Jha MK, Matza D, Flavell RA (2013). Emerging roles of L-type voltage-gated and other calcium channels in T lymphocytes. Front Immunol..

[19] Yeromin AV, Zhang SL, Jiang W, Yu Y, Safrina O, Cahalan MD (2006). Molecular identification of the CRAC channel by altered ion selectivity in a mutant of Orai. Nature.

[20] Maruyama T, Kanaji T, Nakade S, Kanno T, Mikoshiba K (1997). 2APB, 2-aminoethoxydiphenyl borate, a membrane-penetrable modulator of Ins(1,4,5)P3-induced Ca^2+^ release. J Biochem.

[21] Johenning FW, Theis AK, Pannasch U, Ruckl M, Rudiger S, Schmitz D (2015). Ryanodine receptor activation induces long-term plasticity of spine calcium dynamics. PLoS Biol.

[22] Seo MD, Enomoto M, Ishiyama N, Stathopulos PB, Ikura M (2015). Structural insights into endoplasmic reticulum stored calcium regulation by inositol 1,4,5-trisphosphate and ryanodine receptors. Biochim Biophys Acta.

[23] Baba Y, Matsumoto M, Kurosaki T (2014). Calcium signaling in B cells: regulation of cytosolic Ca^2+^ increase and its sensor molecules, STIM1 and STIM2. Mol Immunol.

